# Supplementary material for: Multi‐dataset identification of innovative feature genes and molecular mechanisms in keratoconus
Source: J Cell Mol Med. 2024 Sep 19;28(18):e70079. doi: 10.1111/jcmm.70079 (PMC11412914; doi:10.1111/jcmm.70079)
Supplement: Supplementary file 2 — Material S2. [file JCMM-28-e70079-s002.zip › 7.GSEA analysis/1.ARL11/KEGG_BASAL_TRANSCRIPTION_FACTORS.html]

Details for gene set KEGG\_BASAL\_TRANSCRIPTION\_FACTORS[GSEA]

|  || Dataset | ARL11.group.cls#h\_versus\_l |
| Phenotype | group.cls#h\_versus\_l |
| Upregulated in class | l |
| GeneSet | KEGG\_BASAL\_TRANSCRIPTION\_FACTORS |
| Enrichment Score (ES) | -0.26914507 |
| Normalized Enrichment Score (NES) | -1.1363727 |
| Nominal p-value | 0.31337327 |
| FDR q-value | 1.0 |
| FWER p-Value | 0.993 |
Table: GSEA Results Summary

  

Fig 1: Enrichment plot: KEGG\_BASAL\_TRANSCRIPTION\_FACTORS      
 Profile of the Running ES Score & Positions of GeneSet Members on the Rank Ordered List

  

| SYMBOL | TITLE | RANK IN GENE LIST | RANK METRIC SCORE | RUNNING ES | CORE ENRICHMENT || 1 | TBPL2 | na | 3636 | 0.214 | -0.0002 | No |
| 2 | GTF2A1 | na | 8471 | 0.083 | -0.0987 | No |
| 3 | TAF5L | na | 9863 | 0.062 | -0.1085 | No |
| 4 | GTF2E1 | na | 10432 | 0.055 | -0.0982 | No |
| 5 | TAF6L | na | 10900 | 0.049 | -0.0879 | No |
| 6 | STON1 | na | 11603 | 0.040 | -0.0884 | No |
| 7 | TAF2 | na | 11834 | 0.038 | -0.0765 | No |
| 8 | TAF1 | na | 12976 | 0.027 | -0.0961 | No |
| 9 | TAF4 | na | 13402 | 0.024 | -0.0967 | No |
| 10 | GTF2I | na | 15622 | 0.006 | -0.1575 | No |
| 11 | TBP | na | 15760 | 0.005 | -0.1588 | No |
| 12 | TBPL1 | na | 16236 | 0.002 | -0.1715 | No |
| 13 | TAF1L | na | 16818 | -0.002 | -0.1872 | No |
| 14 | TAF6 | na | 16840 | -0.002 | -0.1867 | No |
| 15 | TAF12 | na | 17125 | -0.004 | -0.1929 | No |
| 16 | TAF7 | na | 19721 | -0.022 | -0.2570 | No |
| 17 | GTF2A2 | na | 20144 | -0.025 | -0.2571 | Yes |
| 18 | TAF10 | na | 20222 | -0.025 | -0.2469 | Yes |
| 19 | GTF2H1 | na | 20649 | -0.029 | -0.2452 | Yes |
| 20 | TAF11 | na | 21270 | -0.033 | -0.2467 | Yes |
| 21 | TAF13 | na | 21705 | -0.037 | -0.2412 | Yes |
| 22 | GTF2E2 | na | 22158 | -0.041 | -0.2339 | Yes |
| 23 | GTF2F2 | na | 22886 | -0.049 | -0.2310 | Yes |
| 24 | GTF2H3 | na | 23197 | -0.052 | -0.2143 | Yes |
| 25 | TAF9 | na | 23417 | -0.055 | -0.1938 | Yes |
| 26 | GTF2F1 | na | 24208 | -0.065 | -0.1846 | Yes |
| 27 | GTF2IRD1 | na | 24742 | -0.073 | -0.1645 | Yes |
| 28 | GTF2H4 | na | 24926 | -0.075 | -0.1330 | Yes |
| 29 | GTF2B | na | 25086 | -0.077 | -0.0999 | Yes |
| 30 | TAF9B | na | 25213 | -0.079 | -0.0649 | Yes |
| 31 | TAF4B | na | 30977 | -0.220 | -0.1235 | Yes |
| 32 | TAF7L | na | 31159 | -0.226 | -0.0180 | Yes |
| 33 | TAF5 | na | 31671 | -0.247 | 0.0881 | Yes |
Table: GSEA details [plain text format]

  

Fig 2: KEGG\_BASAL\_TRANSCRIPTION\_FACTORS      
 Blue-Pink O' Gram in the Space of the Analyzed GeneSet

  

Fig 3: KEGG\_BASAL\_TRANSCRIPTION\_FACTORS: Random ES distribution      
 Gene set null distribution of ES for **KEGG\_BASAL\_TRANSCRIPTION\_FACTORS**
